# A Comprehensive Review of Protein Biomarkers for Invasive Lung Cancer

**DOI:** 10.3390/curroncol31090360

**Published:** 2024-08-23

**Authors:** Alexandre Mezentsev, Mikhail Durymanov, Vladimir A. Makarov

**Affiliations:** 1 Medical Informatics Laboratory, Yaroslav-the-Wise Novgorod State University, 173003 Veliky Novgorod, Russia; durymanov.mo@novsu.ru (M.D.); vladimir.makarov@novsu.ru (V.A.M.); 2 Center for Theoretical Problems of Physicochemical Pharmacology, 109029 Moscow, Russia

**Keywords:** lung cancer, invasiveness, biomarker, lysyl oxidases, non-canonical MAPKs, periostin, prolyl 4-hydroxylases, integrins, polo-like kinases

## Abstract

Invasion and metastasis are important hallmarks of lung cancer, and affect patients’ survival. Early diagnostics of metastatic potential are important for treatment management. Recent findings suggest that the transition to an invasive phenotype causes changes in the expression of 700–800 genes. In this context, the biomarkers restricted to the specific type of cancer, like lung cancer, are often overlooked. Some well-known protein biomarkers correlate with the progression of the disease and the immunogenicity of the tumor. Most of these biomarkers are not exclusive to lung cancer because of their significant role in tumorigenesis. The dysregulation of others does not necessarily indicate cell invasiveness, as they play an active role in cell division. Clinical studies of lung cancer use protein biomarkers to assess the invasiveness of cancer cells for therapeutic purposes. However, there is still a need to discover new biomarkers for lung cancer. In the future, minimally invasive techniques, such as blood or saliva analyses, may be sufficient for this purpose. Many researchers suggest unconventional biomarkers, like circulating nucleic acids, exosomal proteins, and autoantibodies. This review paper aims to discuss the advantages and limitations of protein biomarkers of invasiveness in lung cancer, to assess their prognostic value, and propose novel biomarker candidates.

## 1. Introduction

Cancer is a life-threatening condition with the outlook for patients deteriorating as the disease progresses to metastasis, the process by which cancer cells move away from the primary tumor to colonize distant organs. During tumorigenesis, these cells acquire unique capabilities usually not exhibited by their healthy counterparts [1]. They become penetrative and immortalized. They divide indefinitely and become self-sufficient for mitogenic signals. They also tune up the regulatory mechanisms of the cell cycle to avoid cell cycle arrest and prevent apoptosis [2]. Residing inside a growing tumor, they experience genetic and epigenetic alterations that let them induce angiogenesis, settle in the neighboring tissues, and colonize distant organs [1].

Being one of the most significant health threats worldwide, cancer is responsible for approximately 10 million deaths globally [3]. Among various cancers, lung cancer is a leading cause of death, causing more deaths than prostate, colon, pancreas, and breast cancers combined [4]. The classification of World Health Organization (WHO) distinguishes five types of lung cancer: adenocarcinoma (LUAD), squamous cell carcinoma (LSCC), small cell carcinoma (SLCC), large cell carcinoma (LCC), and other types of lung cancer [5]. All of them, except SLCC and carcinoids, fit the definition of non-small cell lung cancer (NSCLC). LUAD is the largest subtype of non-small cell lung cancer (NSCLC) being accountable for 85% of all lung cancer cases. LUAD originates from the bronchioalveolar stem cells or type I and type II pneumocytes residing in the sub-segmental airways [6]. In healthy lungs, these cells are normally quiescent. However, they start to proliferate and accumulate the mutations undergoing a transformation toward a malignant phenotype [7]. The primary tumors of LUAD often reside in smaller airways, such as the alveoli, located on the outer edges of the lungs. Previous studies suggest that LUAD is more common in non-smokers, and it more frequently affects women and younger individuals under the age of 45 [8].

The TNM classification of lung tumors is a standardized system that describes the extent of tumors, guiding treatment decisions and predicting patient outcomes. Developed in the 1940s, it has undergone several revisions to refine its accuracy. The system stages tumors based on three key factors. The T classification describes the primary tumor, while the N and M classifications describe the pattern of lymph node involvement and distant metastasis, respectively. The combination of these three factors determines the overall stage of lung cancer, ranging from Stage I (early-stage cancer) to Stage IV (advanced cancer with distant metastasis). Unfortunately, many lung cancer cases are diagnosed at an advanced stage, which can significantly impact treatment options and patient outcomes. For instance, in mainland Russia, approximately 34.3% of lung cancer cases are diagnosed at Stage IV, when the cancer has already spread to distant parts of the body [9,10].

Biomarkers are the biomolecules detectable in a patient’s tissues, blood, and other body fluids and dysregulated in certain pathological conditions compared to similar samples of healthy individuals [11]. The possible causes of alterations in the expression of biomarkers include genetic mutations, changes in gene expression, or posttranslational modifications. They come in various forms, such as proteins, nucleic acids, antibodies, and peptides [12]. Some biomarkers are assessable through non-invasive techniques [12], while others require a biopsy or imaging analysis.

Using biomarkers in clinical practice helps us understand the relationship between human biology, diseases, and environmental factors for early detection, diagnosis, and treatment. The three main classes of biomarkers are diagnostic, predictive, and prognostic [13]. Diagnostic biomarkers confirm or reject the diagnosis and help monitor the disease. These biomarkers can be negative or positive depending on their presence in the examined sample. For instance, lung tumors usually express CK7/KRT7 (cytokeratin 7)—the positive predictive biomarker—and do not express CK20/KRT20 (cytokeratin 20)—the negative biomarker. The former helps identify an epithelial tumor, while the latter distinguishes lung and pulmonary metastatic tumors [14].

Predictive biomarkers allow us to assess how the disease will progress and how the patient will respond to the therapy. For instance, commonly used predictive biomarkers for lung cancer are deletions in exon 19 of *EGFR* (epidermal growth factor receptor) and substitution of Leu858 with Arg (exon 21). The other predictive biomarkers are fusion genes (e.g., *EML4*-*ALK*), the mutations in *KRAS* (Kirsten rat sarcoma virus gene), *ERBB2/HER2* (v-erb-b2 avian erythroblastic leukemia viral oncogene homolog 2/human epidermal growth factor receptor 2), *PDL1* (programmed death-ligand 1), etc. [15]. Using this class of biomarkers helps identify the patients who will likely respond to a specific therapy, optimize the treatment regimen, and minimize toxicity. For instance, the mentioned mutations in *EGFR* predict sensitivity to tyrosine kinase inhibitors, such as gefitinib, erlotinib, and afatinib [16].

Prognostic biomarkers assess the clinical outcome and specific time frames, such as overall survival—OS, and event-free survival—EFS. For instance, the expression of *LOXL2* (Lysyl Oxidase Like 2) is the negative prognostic biomarker for many cancers. In patients diagnosed with lung SCC, a high expression of *LOXL2* is associated with a poor prognosis [17]. Because LOXL2 accumulates in various cellular compartments, such as the nucleus and EPR, it disrupts the metabolic and signaling pathways, such as glycosylation and gene expression (see Section 5.5). It also makes LOXL2 a difficult target for anticancer therapy. After all, using predictive biomarkers helps us make more informed decisions about therapy, ensuring that the patients receive an effective and targeted treatment, minimizing toxicity, and improving the overall outcome.

It has become evident that screening tests save lives. Respectively, there are several approaches for detecting lung cancer, such as lab tests, imaging analysis, and biopsy. Protein biomarkers, like carcinoembryonic antigen (CEA) [18], cytokeratin 19 fragment CYFRA 21-1 [19], neuron-specific enolase (NSE) [20], and others, help detect cancer cells in blood or sputum, making these body fluids non-invasive diagnostic tools. However, these biomarkers may also indicate non-cancerous conditions (e.g., CEA is elevated in patients with benign tumors [21], SARS-CoV-2 [22], COPD [23], etc.), and their discovery in body fluids suggests more detailed examination and additional lab tests. Moreover, CEA levels can indicate the response to chemotherapy and be used to monitor the therapeutic response and disease progression [24]. Some biomarkers predict the response to specific treatments, such as EGFR inhibitors (e.g., erlotinib [25], gefitinib [26]) or ALK inhibitors (e.g., crizotinib [27]). Monitoring the specific antigens, like PD-L1, helps analyze the therapeutic response to immunotherapies, such as checkpoint inhibitors (e.g., nivolumab [28], pembrolizumab [29]). In addition, the high efficiency of protein biomarkers led to the development of commercial testing systems [30,31,32] proteomic and antibody arrays for the detection of cancers [33,34].

Academic and clinical scientists have increasingly recognized the potential benefits of proteomic approaches, which include mass spectrometry and alternative technologies [35]. Established protein biomarkers for lung cancer are highly effective in diagnosing the disease, monitoring the response to therapy, and evaluating treatment outcomes [36,37]. The development of machine learning techniques has significantly expanded the possibilities in this field [38,39]. The proposed computer models analyze an extensive array of parameters (features) to classify high-throughput data derived from large cohorts of patients. Recent publications have numerous examples describing the successful integration of machine learning algorithms into biomarker research [40,41]. In the future, the incorporation of these innovative methods into broader interdisciplinary projects led by consortia of researchers will be a common practice. These comprehensive studies will link multi-omic data with the results obtained by traditional methods, thereby optimizing the selection of treatment options for each patient to achieve the best possible outcome.

Specific biomarkers for invasive cancer cells become detectable when tumor cells reach a specific state in their development [42]. Early detection of cancer invasiveness determines the likelihood of metastasis, which is a critical factor for prognosis and assessing a treatment strategy for cancer patients [43]. For this reason, there is a high demand for protein biomarkers that can help diagnose and monitor lung cancer in clinical settings [44]. Some of these biomarkers, such as LOX (lysyl oxidase), LOXL2, MDM2 (mouse double minute 2 homolog), CK7, TTF1, MAPK15/ERK8 (mitogen-activated protein kinase 15), and POSTN (periostin), are already being used for this purpose. In the future, non-protein biomarkers discovered among exosomes, microRNAs, and circulating nucleic acids will fulfill their list to benefit cancer patients [45]. In this review paper, we aim to evaluate the protein biomarkers associated with invasive phenotype in lung cancer, discuss their role in tumorigenesis, and propose novel candidate biomarkers.

## 2. Methods

This review aimed to analyze the literature on identified and potential protein biomarkers for invasive lung cancer. The paper evaluated the impact of individual biomarkers on cell motility and epithelial–mesenchymal transition (EMT), as well as their role in invasive lung cancer. The assessment of protein biomarkers recommended for lung cancer was carried out, with a critical review of the quality of the chosen studies. The authors primarily focused on papers published between 2019 and May 2024 available from PubMed and Web of Science. The search results were supplemented by a thorough manual search of the literature using references from retrieved articles and the Google website. The existing literature on this topic, including in vitro, animal, and human studies, was taken into account. Retracted papers, and papers with questionable experimental design or insufficient experimental evidence were excluded.

The process began with an initial search query for the terms “lung”, AND “invasion”, AND “biomarker”. The extracted data were divided into equal parts, analyzed, and then exchanged within the group for discussion at a joint meeting to agree on the list of biomarkers. Following this, a second search was conducted using the terms “name of suggested biomarker” and “cancer”. The retrieved publications were then assigned to co-authors for evaluation after being split by subject and divided into equal parts. During the review process, we assessed whether suggested biomarkers had the potential to contribute to the invasive phenotype and provided experimental evidence of their involvement. Results were shared and discrepancies were resolved through discussion at a joint meeting, before incorporation of the drafts into the manuscript along with illustrations. The corresponding author wrote the final version of the paper and edited the text. A total of 244 articles were cited in the main text. Additional papers were suggested by reviewers or added by us during the peer review process to respond the reviewers’ comments.

## 3. Early Stages of Tumorigenesis

Tumorigenesis is a complex process that starts with genetic alterations promoting the proliferation of cells with a higher proliferation rate [46]. Some cells lose control over the cell cycle due to mutations targeting specific checkpoints. These mutations impair apoptosis and the repair of DNA. Then, these cells override the Hayflick limit by reactivating telomerase (TERT). Notably, these striking events take place in a relatively small area. The cells compete with each other during clonal selection [47]. A large number of them still undergo apoptosis and die [48]. However, their more resilient counterparts find ways to cope with their challenging conditions. For instance, the benign tumor produces and secretes proangiogenic cytokines that promote the migration and proliferation of vascular cells, leading to angiogenesis and neovascularization of the tumor [48]. Supplying the tumor with oxygen and nourishment, the new blood vessels also act as an escape route for malignant cells that do not have enough space, pushing them to disseminate and colonize distant organs [49].

## 4. Epithelial–Mesenchymal Transition of Tumor Cells and Dissemination of Cancer

In the early stages of tumorigenesis, the cells forming an epithelial tumor are not mobile enough to spread cancer. However, during EMT, the affected cells undergo morphological changes and stop dividing. The tumor cells undergoing EMT change shape by elongating and forming protrusions [50]. The cell polarity also changes from apical–basal to front–rear [51]. The principal changes occur inside the tumor cells. In the cytoplasm, the faster-growing microtubules exhibit a radial arrangement [52]. The actin stress fibers become co-aligned along the longitudinal cell axis, and the focal adhesions decrease in size but increase in number. The nucleus adjusts its shape and size and also becomes elongated. The cells undergo fundamental changes in gene expression. They also experience a reorganization of chromatin and alteration in the nuclear architecture [53]. The nuclear envelope, enclosing the nucleus, undergoes modifications in structure and composition, impacting nuclear–cytoplasmic interactions and signaling pathways that regulate cell morphology and migration [54]. As a result, the transformed cells gain motility and acquire an invasive phenotype.

It is important to note that not all tumor cells undergo EMT at the same time. Previous studies have shown that cells with either an epithelial or mesenchymal phenotype are present in the center of the tumor (e.g., [55]) and cooperate by establishing intercellular contacts and exchanging signals. Some of these signals help the epithelial cells to initiate EMT and prepare for invasion. Meanwhile, the mesenchymal phenotypic cells are more common in the border region of tumor [56].

The conversion from epithelial to mesenchymal state is not necessarily complete in either primary or secondary epithelial tumors [57]. Many cells acquire an intermediate phenotype (p-EMT/partial EMT). These cells behave similarly to mesenchymal-like tumor cells. Expressing both epithelial and mesenchymal markers, they preserve their tumorigenicity and the power to establish the metastatic colonies [58]. Contrarily, the cells completed EMT are less tumorigenic. However, they are mainly responsible for drug resistance.

Acquiring the invasive phenotype, some cells establish colonies within the damaged organ, while others penetrate nearby blood and lymphatic vessels and become circulating tumor cells (CTCs) [59]. These CTCs travel to lymph nodes and distant organs, continuing to express mesenchymal biomarkers, indicating that they can help monitor the disease flow [60]. CTCs are also known as tumor-initiating cells (TICs) or tumorigenic cells [61] for their ability to self-renew and constitute the tumor. At a new place, CTCs undergo a mesenchymal-to-epithelial transition (MET), where they regain the epithelial phenotype, start dividing, and form colonies, also known as metastases or metastatic nodules [62].

Being a complex phenomenon, EMT combines multiple well-coordinated events necessary to give rise to the outgrowth of metastatic tumors in a new organ environment [63]. Its smooth transition at the mRNA level requires coordination by a dozen transcription factors, including zinc-finger proteins SNAI1/2 and ZEB1/2 (snail family transcriptional repressors 1/2 and zinc finger E-box-binding homeobox and zinc finger E-box-binding homeobox 1), twist family basic helix-loop-helix transcription factors TWIST1/2, and AP1 transcription factors c-FOS and FOSL1 (AP1- activator protein 1; c-FOS and FOSL1—FOS proto-oncogene and FOS-like 1 AP-1 transcription factor subunit, respectively) [64,65,66]. Moreover, several microRNAs (miRNAs), like miR-200 and miR-128, contribute to EMT by forming double-negative feedback loops. These miRNAs also regulate the expression of the B lymphoma Mo-MLV insertion region 1 homolog (BMI1) protein, which positively regulates the expression of the EMT marker SNAI1 [67]. In addition, non-coding RNAs regulate the translation of mRNAs and chromatin remodeling [68]. Tumor-associated cells also contribute to EMT by release of signaling molecules, such as cytokines. For instance, cancer-associated fibroblasts (CAFs) release CXCL12 (C-X-C motif chemokine ligand 12) to facilitate EMT by cancer cells [69]. By activating collagen receptors on the cell surface, CAFs also induce the key genes/transcription factors of EMT mentioned above [70].

## 5. Biomarkers for Invasive Lung Cancer

Presently, the clinical use of CTC specific biomarkers to detect the invasiveness of cancer has certain limitations [71]. Most potential biomarkers expressed in CTCs are shared with resident stem cells (RSCs), embryonic stem cells (ESCs), or differentiated cells [72]. They often label heterogeneous cell populations, whereas individual populations of cells would be easier to identify and characterize using combinations of several biomarkers rather than individual proteins. For instance, a well-established biomarker for hematopoietic stem and progenitor cells, CD34, is detectable in other types of cells, namely interstitial cells, endothelial cells, fibrocytes, and muscle satellite cells [73]. Some of them are recruitable to the tumor microenvironment, while others, like healthy lung cells residing in the bordering area, are not [74]. The expression of CD34 is greater in lung tumors [73,74]. However, it also occurs in adjacent healthy lung tissue [74] and malignant tumors of different origin [75]. In this regard, even double staining on CD34 and PLAUR may cause a misidentification of metastatic and non-metastatic LUADs since the immunostaining for CD34 reveals angiogenesis in tumors of different origins [75]. Due to the limited specificity of CTC-specific biomarkers, we propose considering proteins associated with EMT and post-EMT that are directly involved in the motility of cancer cells for experimental and clinical studies of invasive lung cancer. (Table 1).

### 5.1. Cytokeratin 7

Cytokeratin 7 (KRT7) is one of the cytokeratins residing in simple epithelia, including the lung epithelium. LUAD tumors aberrantly overexpress KRT7. Moreover, KRT7 is one of the few cytokeratins whose expression increases in cancer cells during EMT [76]. The expression of *KRT7* correlates with the tumor growth and metastasis and the levels of the EMT-associated transcription factors (SNAI1, SNAI2, TWIST1, TWIST2), and the mesenchymal markers, such as fibronectin, and vimentin (*VIM*) [76]. Cancer cells with high levels of KRT7 are less susceptible to apoptosis. They exhibit a higher proliferation rate and motility [77]. In addition, the aberrant expression of *KRT7* in lung cancer cells could make it a potential diagnostic biomarker for the disease.

In a recent study [78], Zhao et al., revealed that a high level of KRT7 indicates a deficiency of KRT7-AS lncRNA in malignant cells. In healthy cells, this lncRNA targets *KRT7* mRNA for degradation, and the level of KRT7 is low. Contrarily, suppression of KRT7-AS lncRNA in cancer cells causes an accumulation of KRT7, and the level of KRT7 increases. This regulatory mechanism plays an essential role in tumorigenesis due to the ability of KRT7-AS lncRNA to interact with the tumor suppressor protein PTEN. Binding to PTEN, this lncRNA prevents its ubiquitination and following degradation in proteasomes. In turn, repressing KRT7-AS lncRNA allows the cancer cells to lower the level of PTEN and minimize its antitumorigenic effects. However, another consequence of lowering KRT7-AS lncRNA is the prevention of KRT7 mRNA from degradation and, respectively, its upregulation in malignant cells that we observe.

High levels of KRT7 would help identify the subtypes of lung cancer or predict patient prognosis [79], guiding personalized treatment strategies. The immunostaining for KRT7 of tumors and CTCs may help diagnose lung cancer and optimize the treatment strategy for individual patients. The previous studies demonstrated high levels of KRT7 in aggressive lung tumors that tend to invade and disseminate [76]. In lung cancer, the upregulation of *KRT7* mRNA indicated the presence of CTCs among the peripheral blood cells [80]. Using the blood samples drawn from healthy donors, the authors showed a strong correlation between the level of *KRT7* mRNA and the number of A549 cells added to the blood. However, they did not suggest using *KRT7* mRNA for monitoring the therapeutic response to the chemotherapeutic drugs or survival rate in patients with advanced lung cancer because CTCs appeared to be significantly more resistant to chemotherapeutic drugs than non-metastatic tumor cells. In addition, using KRT7 alone for distinguishing lung and other cancers would not be enough since this protein is detectable in metastatic tumors of different origins [77].

### 5.2. Cytokeratin 20

The expression level of Cytokeratin 20 (KRT20) is often helpful as a biomarker for identification and distinguishing between different types of cancer. In the body, the highest expression of KRT20 is in intestinal gland crypt cells and cutaneous Merkel cells [80]. KRT20 possesses some characteristics of immunophenotype, which are critical for the differential diagnosis of pulmonary primary LUAD and morphologically similar metastatic tumors of different origins [81]. The LUAD tumors are essentially KRT20-negative [82], whereas others, like colorectal and prostate carcinomas, are usually KRT20-positive [83]. The previous research suggests that KRT20 may serve as a potential biomarker for diagnosing and monitoring lung cancer to distinguish them from KRT20-positive tumors. Thus, the immunostaining for KRT20 in tumors or CTCs could be helpful in early detection and adjustments of personalized treatment strategies for lung cancer patients.

### 5.3. TTF1

Thyroid transcription factor 1 (TTF1) is a member of the NKX2 family of homeodomain transcription factors [84]. In the body, TTF1 is predominantly located in the nuclei of type II pneumocytes and Clara’s cells (non-ciliated epithelial cells found in the terminal bronchioles of the lungs), thyroid tissues, and the diencephalons of the brain [85]. In the lung, TTF1 binds to and helps to regulate the secretory activity of Clara’s cells and the expression of pulmonary surfactant proteins [86]. The expression of TTF1 increases after stimulation with fibroblast growth factor, bone morphogenetic proteins (BMPs), or sonic hedgehog factor [84]. In addition, TTF1 participates in the development of bronchiolar epithelial cells and plays a role in protecting the respiratory epithelium from injuries and infections.

According to the previous studies, TTF1 plays an active role in lung cancer [87]. In many cases, there is a significant amplification on the TTF1 gene locus in the genome. In cultured LUAD cells, it increases the proliferation rate [88] and cell viability [89]. The other lung tumors, like malignant mesotheliomas and squamous cell carcinoma (SCC) tumors, remain TTF1-negative [85]. The immunostaining for TTF1 alone does not distinguish between pulmonary adenocarcinomas and adenocarcinomas of non-pulmonary origin because ~75–80% of them are TTF1-positive [90]. However, most LUAD tumors have the phenotype TTF1^+^/CK7^+^/CK20^−^. Respectively, the differential diagnostic of primary adenocarcinomas originating from lung cells from metastatic adenocarcinomas made of the malignant cells traveling from distant organs and tissues requires the panel of three antibodies directed to CK7, CK20, and TTF1 [91]. In this case, the specificity of detection is close to 100%.

### 5.4. Lysyl Oxidase

**Figure 1:**
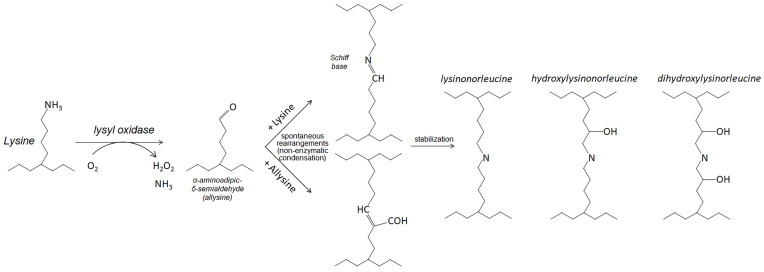
Oxidative deamination catalyzed by lysyl oxidases. In the beginning (left), the enzyme catalyzes the oxidative deamination of a lysyl residue in a targeted protein substrate. The product of the enzyme reaction (allysine/peptidyl-α-aminoadipic-δ-semialdehyde) participates in subsequent nonenzymatic condensation reactions. The interactions with lysine and allysine yield the formation of bifunctional crosslink intermediates, Shiff base, and aldol (upper and lower intermediates in the middle). The further spontaneous rearrangements lead to formation of tri-, tetra-, and even penta-functional crosslinks in the affected proteins.

LOX is an enzyme crosslinking collagen and elastin fibers in connective tissues [92]. Oxidizing their lysine residues, LOX generates stable covalent bonds between these proteins (Figure 1). Crosslinking collagen and elastin provides strength and elasticity to various tissues in the body, such as skin, blood vessels, tendons, and ligaments. The other catalytic byproducts, hydrogen peroxide (H_2_O_2_) and ammonia, generate reaction oxygen species (ROS) and signal transduction. LOX requires a proteolytic activation [93].

The major producers of LOX in the body are fibroblasts, smooth muscle cells, and other cells of connective tissues [94]. In the healthy body, LOX plays an active role in tissue remodeling and wound healing [95]. LOX also contributes to the structural rearrangements of the tumor microenvironment (TME) by surrounding the tumor with a dense layer of ECM proteins [96]. The increased crosslinking of collagen fibers results in a stiffer ECM, which creates hypoxic conditions in the tumor bedding and promotes angiogenesis. In hypoxic conditions, tumor cells increase the secretion of angiogenic factors that promote the growth of new blood vessels in the surrounding tumor area, encouraging the cancer cells to disseminate and metastasize. Upregulation of LOX also improves the resistance to anticancer therapeutic agents by preventing their penetration to the inner area of the tumor [97].

**Figure 2:**
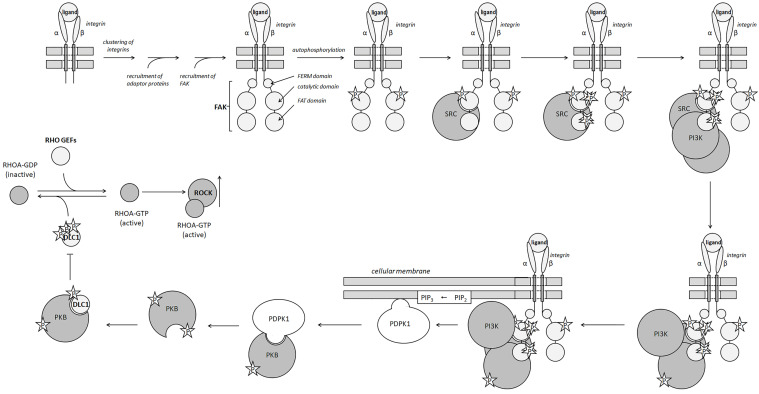
Activation of the PI3K/PKB and RHO/ROCK pathways by integrins. Activated integrin receptor initiates signal transduction by binding to the tyrosine-protein kinase, FAK. The binding of FAK follows clustering the integrins and the recruitment of adaptor proteins. In response to the interaction with the ligand–receptor complex, FAK autophosphorylates. The autophosphorylation of FAK causes conformational changes and creates a binding site for SRC kinase. After engaging FAK, SRC becomes activated and phosphorylates few additional amino acids. The activated SRC kinase recruits phosphoinositide 3-Kinase (PI3K) to the complex and phosphorylates it. PI3K phosphorylates the 3-position hydroxyl group of the inositol ring of PIP_2_, a small lipid anchored in the cellular membrane, and converts it to PIP_3_. Traveling in the inner layer of the membrane, PIP_3_ interacts with PDPK1. Binding PIP_3_ anchors PDK1 in the cellular membrane. Then, PDK1 recruits PKB and activates it by phosphorylation. One of the PKB target proteins, DLC1 (Deleted in Liver Cancer 1), is a GTPase-activating protein that inactivates RHOA by stimulating the hydrolysis of GTP bound to the enzyme. Phosphorylating DLC1, PKB suppresses this biological effect and shifts the balance toward RHOA-GTP, which activates ROCK by causing conformational changes in its molecule.

Significant changes in the extracellular environment, like hypoxia, dysregulate the expression of LOX [98]. The aberrant expression and accumulation of LOX is common for various types of cancer. In these cells, the intracellular LOX activates TGFβ (transforming factor β) and HIF1α (hypoxia-inducible factor 1α) signaling pathways [99]. Moreover, the extracellular LOX produces the same effect by transducing the signal to downstream FAK/SRC via INT surface receptors of the affected cells [100]. The activated TGFβ and HIF1α signaling pathways, in turn, activate the EMT essential transcription factors (SNAI1, SNAI2, etc.). In this regard, LOX stabilizes HIF1α in an H_2_O_2_-dependent manner [101]. Then, HIF1α upregulates SNAI1 by binding to a hypoxia-response element (HRE) element in the LOX promoter [102]. Additionally, LOX promotes EMT by enhancing the PI3K/PKB signaling pathway (PI3K—phosphoinositide 3-kinase and PKB—protein kinase B); Figure 2 [99].

In turn, the extracellular LOX modifies the ECM proteins, promoting the formation of focal adhesions and the activation of integrins. The β-integrins (INTβ)—specifically INTβ1, INTβ3, and INTβ4—play the role of mechanosensors. Their activation recruits non-receptor tyrosine kinase SRC (SRC proto-oncogene, non-receptor tyrosine kinase) and the consequent phosphorylation of focal adhesion kinase (FAK) [103]. In turn, phosphorylated FAK activates the RHO/ROCK signaling pathway (Figure 2), which causes the remodeling of the actin cytoskeleton and the acquisition of a migratory phenotype [104].

A significant increase in the expression of LOX in early LUAD coincides with the infiltration of the primary tumor by immune cells [105]. Then, the level of LOX keeps growing as the disease progresses to the advanced stages [106]. Moreover, patients with advanced LUAD also exhibit high levels of LOX in the serum, which indicates metastasis of lymph nodes and distant metastasis. The level of LOX also indicates a correlation with the TNM (tumor–node–metastasis, a widely used classification to describe the extent of cancer) stage of the disease [107]. For these reasons, some authors propose LOX as a biomarker for assessing the invasive potential of the primary tumors and recommend using the high level of LOX as an independent factor of poor prognosis even in the early stages of LUAD [108]. According to the published assessments, the 5-year survival rates for patients with low and high levels of LOX are 71 and 43%, respectively [106]. These assessments are in line with the data of experimental studies. For instance, the knockdown of *LOX* in human LUAD cells represses their invasion and migration [109], suggesting that specific inhibitors of LOX would be capable of slowing down tumor growth and metastasis in LUAD patients [108].

### 5.5. LOXL2

Lysyl oxidase-like 2 (LOXL2) is another secreted copper-dependent amine oxidase with a role similar to LOX [110]. Since LOXL2 has a lysyl oxidase activity (Figure 1), it covalently crosslinks collagen and elastin fibers of ECM via the oxidative deamination of ε-amino groups of lysines. LOXL2 also produces hydrogen peroxide and ammonia as byproducts of the catalytic process to generate ROSs and activate the FAK pathway [111].

**Figure 3:**
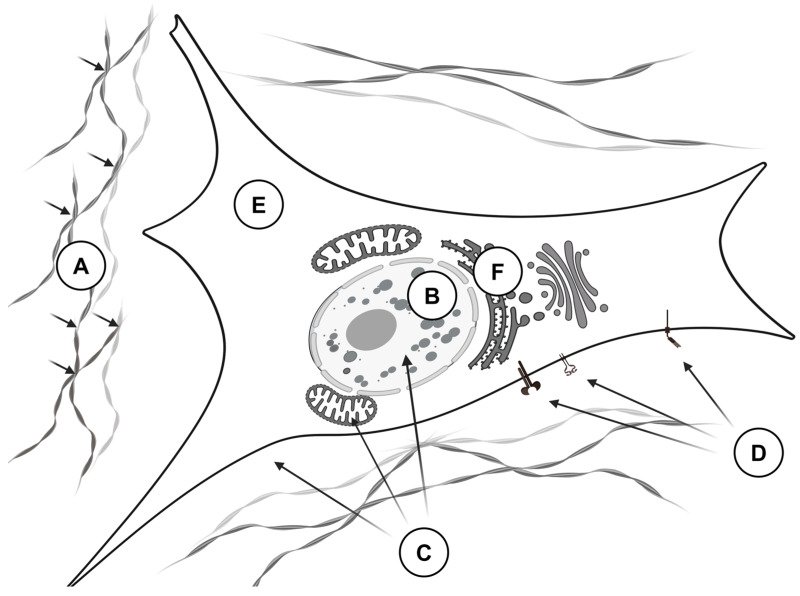
The biological effects of LOXL2 that promote the invasive behavior of tumor cells. LOXL2 exerts its effects through multiple mechanisms: (**A**)—In the extracellular matrix, LOXL2 crosslinks proteins such as collagens, elastins, and fibronectins, leading to the formation of a dense and rigid matrix that facilitates tumor cell invasion (small black arrows indicate the crosslinking sites). (**B**)—In the nucleus, LOXL2 deaminates histones H3 and other DNA- and RNA-binding proteins (including SNAI2, TCF3, and KLF4), resulting in chromatin condensation and altered gene expression. (**C**)—In the cytoplasm and the nucleus, LOXL2 generates reactive oxygen species (ROS) that cause protein and DNA damage, contributing to genomic instability. (**D**)—LOXL2 modulates intracellular signaling by oxidizing lysine residues on cellular receptors, such as PDGFRβ, thereby activating the downstream signaling pathways. (**E**)—In the cytosol, LOXL2 prevents apoptosis by inactivating MARCKSL1 and promotes the Warburg effect by deacetylating fructose-bisphosphate aldolase A. (**F**)—In the endoplasmic reticulum, LOXL2 interferes with protein glycosylation and induces a stress response by interacting with the heat shock protein HSAP5.

Unlike LOX, LOXL2 crosslinks and stabilizes insoluble collagen deposition in tumor tissues (Figure 3) [112]. Moreover, its catalytic domain can deaminate lysine residues in the histone H3 and TAF10 (TATA-Box Binding Protein Associated Factor 10), a subunit of transcription initiation factor 2D (TFIID) [113]. At the N-terminus, LOXL2 contains four scavenger receptor cysteine-rich (SRCR) domains, which participate in interactions of LOXL2 with its protein binding partners [114]. Previous studies demonstrated that SRCR domains of LOXL2 bind ECM proteins, like fibronectin and collagens [115]. Moreover, one of these domains is necessary for interacting with RNA-binding proteins [116]. About a dozen miRNAs (e.g., miR-29 and miR-504) regulate the stability of LOXL2 mRNA at the posttranscriptional level by silencing it in the cytoplasm. In turn, some lncRNAs counteract them. For instance, ROR1-AS1 lncRNA (ROR1 antisense long non-coding RNA 1) interferes with miR-504 in bladder and lung cancer cells [117].

After folding, LOXL2 undergoes posttranslational modifications. For example, the protein has three different sites for N-glycosylation. This posttranslational modification is necessary for secreting LOXL2 from the cells [118]. Cai et al. [119] found that LOXL2 is susceptible to phosphorylation with large tumor suppressor kinase 1 (LATS1) in ovarian granulosa cells. The authors suggest that phosphorylation of LOXL2 is necessary to control the expression of STAR (steroidogenic acute regulatory protein), a protein that facilitates the transportation of cholesterol to the inner mitochondrial membrane. Although they did not verify how this posttranslational modification affects the biological effects of LOXL2, they proposed that the phosphorylation might target LOXL2 for the following ubiquitinylation and lead to its degradation in proteasomes. Although LOXL2 does not require a proteolytic activation [120], it is susceptible to proteolytic cleavage by furin, paired basic amino acid cleaving enzyme 4 (PACE4), or proprotein convertase subtilisin/kexin type 5 (PCSK5). The cleavages occur in the middle of the polypeptide and do not affect the ability of LOXL2 to crosslink collagen and elastin [120]. However, it broadens the substrate specificity of the enzyme, making it possible for LOXL2 to oxidize insoluble collagen IV. In addition, LOXL2 undergoes ubiquitinylation by TRIM44 (tripartite motif-containing protein 44) and the following degradation in proteasomes [121].

LOXL2 is a secreted protein that contributes to remodeling the extracellular matrix (ECM). By crosslinking collagen and elastin fibers, LOXL2 enhances the structural integrity and tensile strength of the ECM. These changes lead to the organization of the collagen fibers into thick and dense bundles, promoting the development of desmoplasia and hypoxic conditions [100]. The appearance of hypoxic regions is crucial for the growth of invasive tumor cells and the expansion of solid tumors. It results in the upregulation of hypoxia-induced genes, such as the proangiogenic factor VEGF (vascular endothelial growth factor), leading to a neovascularization of the tumor, and the progression of tumorigenesis to metastasis [114].

In the cell, hypoxia is one of the main factors controlling the expression of LOXL2 [122]. By binding to a hypoxia response element in intron 1, hypoxia-inducible factor 1 (HIF1) induces the *LOXL2* gene [123]. Moreover, HIF1 increases the expression of *LOXL2* by recruiting the lysine demethylase 4C (KDM4C) to the promoter area where KDM4C demethylates histone H3 [124]. The hypoxic conditions also cause significant readjustments in tumor-associated cells. In M2 macrophages, the changes in TME activate the INTβ5-FAK-MEK1/2-ERK1/2 (MEK—mitogen-activated protein kinase/extracellular signal-regulated kinase kinase; ERK—extracellular signal-regulated kinase) signaling pathway, leading to the stabilization of HIF1 and upregulation of HIF1 target genes, including *LOXL2* [125]. In CAFs, the secreted LOXL2 activates the FAK/PKB signaling pathway via the interaction with INTβ3. This signaling cascade promotes the activation of fibroblasts and induces *LOXL2* [111]. Moreover, LOXL2 stimulates CAFs to secrete pro-lymphatic vascular factor (VEGFC) and stromal cell-derived factor 1 (SDF1) in an HIF1-dependent manner. The named growth factors, in turn, activate SNAI1 and ERK MAPKs in the endothelial cells of lymphatic vessels, enhancing their proliferation and tumor lymphangiogenesis [126]. In addition, the secretion of VEGFA and PDGF (platelet-derived growth factors) by tumor cells induces *LOXL2* in endothelial cells of tumor-associated blood vessels, stimulating angiogenesis to vascularize the tumor [126].

The interaction of LOXL2 with proteins involved in cell adhesion and signaling pathways increases the aggressiveness of tumor cells. By generating ROS as a byproduct of the catalytic process, LOXL2 upregulates ERBB2 [127]. It also oxidizes lysine residues of PDGFRβ located on the surface of stromal fibroblasts. The oxidation improves the outcome of the ERK/MAPK signaling pathway. It also increases the proliferation rate of the affected cells [128]. In turn, the interaction of LOXL2 with myristoylated alanine-rich C kinase substrate-like 1 (MARCKSL1) improves the survival of cancer cells by inhibiting the MARCKSL1-induced apoptosis [129]. In addition, LOXL2 deacetylates fructose-bisphosphate aldolase A (ALDOA). In this case, the deacetylation promotes the relocation of the aldolase from the cytoskeleton to the cytosol, enhancing the catabolism of glucose [130].

In cancer, various external factors and the dysregulation of many intracellular signaling pathways may displace and cause an abnormal expression of *LOXL2*. The accumulation of LOXL2 in the endoplasmic reticulum (EPR) of cancer cells, which occurs in tumors of higher grade, initiates the ERR-stress response due to the interaction of LOXL2 and heat shock 70 kDa protein 5 (HSPA5) [131]. The latter stimulates the IRE1-XBP1 signaling pathway and activates several EMT essential transcription factors (SNAI1, SNAI2, and ZEB2). Their activation, in turn, initiates EMT and stimulates a further secretion of ECM proteins from the cells.

When the infiltration of tumors by cytotoxic CD8-positive T-cells begins, the collagen fibers bind to the leukocyte-associated immunoglobulin-like receptor 1 (LAIR1) on their surface. Their interaction activates protein tyrosine phosphatase (PTPN6)-mediated T cell exhaustion and improves the chances of tumor cells to avoid immune surveillance. Contrarily, the inhibition of LOXL2 reduces the collagen deposits, increases the infiltration of tumors by CD8-positive T-cells, and restores its sensitivity to immune surveillance [132]. In the vascular endothelium, two different mechanisms control the expression of LOXL2. In the extracellular matrix, the secreted epidermal growth factor-like protein 7 (EGFL7), a regulator of vascular elastogenesis, inactivates LOXL2 by binding to its catalytic domain to prevent the conversion of tropoelastin into mature elastin [133] and promote the remodeling of blood vessels [134]. In the nucleus, GATA6-AS lncRNA (antisense transcript of GATA-binding protein 6) prevents the removal of activating H3K4me3 chromatin marks by interacting with the catalytic domain of LOXL2 [135].

Using SRCRs, LOXL2 interacts with various transcription factors (rev. in [121]). The interactions of this kind are unusual for healthy cells because they do not accumulate LOXL2 in the nucleus. However, it occurs in cancer cells that overexpress LOXL2. The interaction of LOXL2 with transcription factors (SNAI2, TCF3/transcription factor 3, KLF4/Krüppel-like factor 4, etc.) plays a crucial role in tumorigenesis because it produces a unique gene-specific response. For instance, binding LOXL2 to TCF3 recruits LOXL2 to the promoter of *CDH1* (the gene encoding E-cadherin) to repress the transcription due to the oxidation of lysine residues of histone H3 [136]. The heteromerization of LOXL2 and SNAI2 improves the stability of SNAI1 by blocking its phosphorylation by glycogen synthase kinase 3 (GSK3β) [137]. The recent experimental studies suggest (rev. in [121]) that LOXL2 uses a similar mechanism to regulate the genes involved in the formation of intercellular contacts, polarity complexes, cell differentiation, etc. In addition, LOXL2 is capable of interacting with chromatin directly, causing its compaction by oxidizing lysine residues [138]. The compaction of chromatin plays a crucial role in cell division of rapidly proliferating cancer cells. It also makes DNA less accessible to the DNA-binding proteins, including ones that perform the damage repair, favoring the accumulation of mutations.

The overexpression of LOXL2 was consistently reported in numerous studies associated with tumor aggressiveness and poor prognosis in various types of cancer [139]. It makes LOXL2 detectable in blood samples of patients with lung SCC, pointing to LOXL2 as a potential biomarker of this form of cancer [17]. Moreover, high levels of LOXL2 are present in tumors of patients with advanced lung cancer. In addition, the upregulation of LOXL2 indicates poor prognosis for patients and the cytoplasmic level of LOXL2 is an independent prognostic factor [140]. These reports link the expression level of LOXL2 and intensive immunostaining for intracellular LOXL2 with worse clinicopathological characteristics of tumors and poor overall survival for patients.

### 5.6. MAPK15

Mitogen-activated protein (MAP) kinase 15 (MAPK15/ERK7—in mice/ERK8—in humans) is one of the atypical MAP kinases that recently emerged as a crucial modulator of autophagy, i.e., a lysosome-dependent degradation of cells, and EMT [141]. The upregulation of *MAPK15* often occurs in the LUAD tumors at the stage when the disease targets lymph nodes. Due to alternative splicing, *MAPK15* mRNA produces three different protein isoforms. Two of them are much shorter than the third one due to truncations of their C-terminal regulatory domain. However, the shorter isoforms preserve the structural integrity of the N-terminal domain and they are also catalytically active [142].

**Figure 4:**
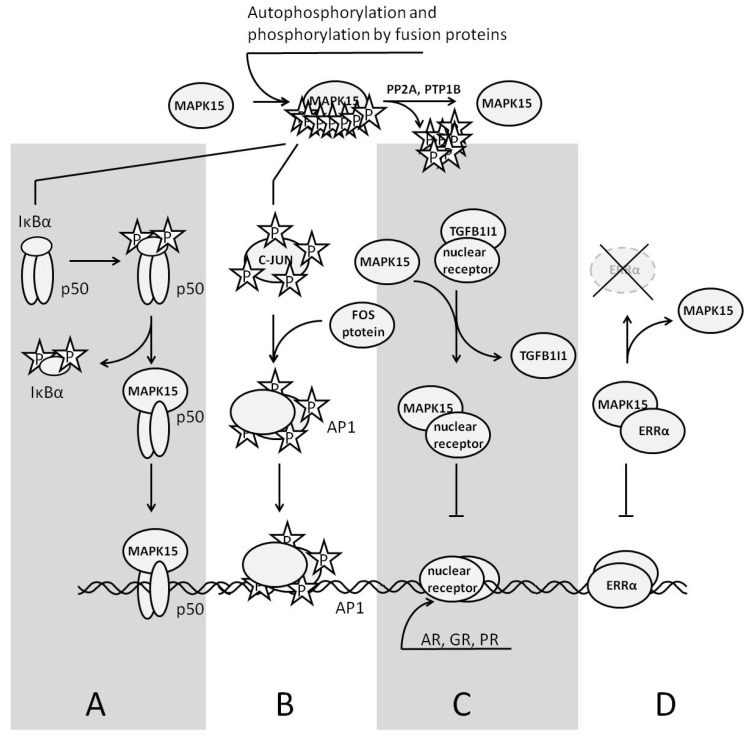
The role of MAPK15 in regulation of gene expression. On the top: two ways to phosphorylate/activate MAPK15—autophosphorylation and phosphorylation by a fusion protein exhibiting a kinase activity; (**A**)—MAPK15 activates the transcription factor NFκB. The enzyme destabilizes the inhibitory subunit IκBα and directly interacts with p50. The complex of p50 and MAPK15 is transcriptionally active; (**B**)—MAPK activates/phosphorylates the AP1 transcription factor c-JUN inducing AP1 target genes; (**C**)—MAPK15 represses the transcription of nuclear receptors (AR, GR, and PR), competing with them for their binding partner, TGFB1I1. (**D**)—MAPK15 represses ERRα target genes by escorting ERRα to the cytoplasm for degradation.

At present, it is unclear whether the expression of MAPK15 occurs during embryogenesis. After birth, the expression of MAPK15 is detectable in various tissues, with higher levels in the lungs and kidneys [143]. No conventional MAPK kinase mediates the phosphorylation/activation of MAPK15 [144]. However, MAPK15 may phosphorylate itself (Figure 4) [145]. In this case, the activated enzyme colocalizes with autophagic structures. It directly interacts with several proteins participating in the autophagic pathway, such as γ-aminobutyric acid receptor-associated protein (GABARAP), GABAA-receptor-associated protein-like 1 (GABARAPL1), and microtubule-associated proteins 1A/1B light chain 3B (MAP1LC3B/LC3B), coordinating the formation of autophagosomes. The overexpression of wild-type MAPK15 increases MAP1LC3B-positive autophagic structures, and silencing the endogenous MAPK15 reduces their number [145].

According to the available experimental data, the activation of MAPK15 follows the treatment of cells with serum [145], damage of the DNA [146], generation of ROS [147], and deprivation of amino acids [148]. In cancer cells, fusion proteins, like BCR-ABL, can directly phosphorylate MAPK15 [149]. On the other hand, two protein phosphatases, namely protein phosphatase 2A (PP2A) and protein-tyrosine phosphatase 1B (PTP1B), dephosphorylating MAPK15 are well known and intensively studied [147]. After activation, MAPK15 translocates to the nucleus and induces some genes, like *JUN* [150].

Heteromerizing with the subunit p50 of the transcriptional factor NFκB (nuclear factor κ-light-chain-enhancer of activated B cells) in cancer cells, MAPK15 translocates to the nucleus. In the nucleus, the complex of p50 and MAPK15 binds to the DNA, changing the expression of some genes and increasing cell motility. As a stress-induced activator, MAPK15 phosphorylates the inhibitory subunit of the transcription factor NFκB, IκBα (nuclear factor of κ light polypeptide gene enhancer in B-cells inhibitor α), promoting its ubiquitinylation and degradation in the proteasomes [151]. MAPK15 also binds to the other transcription factors. Interacting with the transcription factor c-JUN, MAPK15 phosphorylates the same serine residues as c-Jun N-terminal kinase (JNK), which is necessary to promote c-JUN-mediated transcriptional activity [152]. Presumably, this crosstalk between two MAPKs helps to optimize the expression of c-JUN target genes whenever the current status of JNK cannot guarantee the same result.

The biological effects of MAPK15 on nuclear receptors do not require kinase activity. For instance, the activation of MAPK15 downregulates the genes encoding androgen and glucocorticoid receptors (*AR* and *GR*, respectively) by competing with either AR or GR protein for their binding partner, transforming growth factor β1-induced transcript 1 (TGFB1I1) [153]. Binding to estrogen-related receptor α (ERRα) in the nucleus MAPK15 causes the suppression of ERRα target genes by escorting ERRα to the cytoplasm [154]. Contrarily, the loss of function by MAPK15 or its suppression decreases the degradation of ERRα in proteasomes and promotes tumorigenesis in ERRα-positive breast tumors. Since TGFB1I1 also interacts with other nuclear receptors, like progesterone receptor (PR) [155] and PPARγ [156], MAPK15 produces similar effects on their target genes. MAPK15 is also functionally active in other intracellular organelles. In the endoplasmic reticulum, MAPK15 causes the dispersion of SEC16 and the disassembly of the exit sites, interfering with intracellular transport by inhibiting the secretion of cargo in an amino acid-sensitive manner [148]. In the Golgi apparatus, MAPK15 inhibits O-glycosylation [157].

MAPK15 plays a crucial role in the biogenesis of DNA. Binding to a multifunctional protein PCNA (proliferating cell nuclear antigen) essential for replication and repair of DNA, MAPK15 strengthens its interaction with chromatin. Moreover, MAPK15 inhibits the interaction of PCNA and MDM2, which targets PCNA for degradation [146]. Abolishing MAPK15 downregulates *PCNA* in the cell. In turn, the downregulation of *PCNA* leads to cell cycle arrest during the transition from the G_1_ to S phase of the cell cycle. For the same reason, abolishing PCNA impairs the repair of DNA. In turn, a deficiency of MAPK15 in the cell increases the phosphorylation of histone H2AX, although it usually occurs at the ends of DNA double-stranded breaks [146,158]. MAPK15 also activates TERT. According to the previous findings [158], the restoration of the telomeres in cancer cells depends on the presence of MAPK15 and occurs in a kinase-dependent manner.

Elevated expression of *MAPK15* is often seen across various types of cancer and cell lines [159], including lung cancer cells [151]. At the molecular level, the endogenous MAPK15 interferes with the activation of TP53 and prevents the triggering of TP53-dependent mechanisms, and the following cell cycle arrest [160]. Analysis of changes in gene expression suggests that MAPK15 may serve as a biomarker to diagnose lung cancer at early stages and its prognostic assessment. For instance, the expression of *MAPK15* positively correlates with metastasis in lymph nodes in LUAD patients [141].

### 5.7. POSTN

Periostin (POSTN)/osteoblast-specific factor 2 (OSF2) is a secreted matrix N-glycoprotein that lacks a transmembrane domain. POSTN has a high sequence homology with the transforming growth factor β-induced protein (TGFBI), which promotes cell adhesion and the migration of fibroblasts. In the embryo, the expression of *POSTN* occurs in the periosteum and periodontal ligaments, where it plays a crucial role in cardiogenesis [161] and regulates the formation of bone and teeth [162]. In adults, the induction of *POSTN* follows vascular [163], myocardial [164], and some other injuries (rev. in [165]).

The promoter of *POSTN* contains a binding site for the EMT-controlling factor TWIST [166]. Interacting with the promoter, TWIST induces *POSTN*. Moreover, the promoter also contains HRE and responds to the transcription factor HIF1 [167]. In addition, the transcription of *POSTN* intensifies due to the activation of either transforming growth factor α (TGFα) or fibroblast growth factor 2 (FGF2) signaling pathways [165]. The molecule of POSTN contains several domains [168]. The N-terminal signaling peptide necessary for the secretion of POSTN precedes a cysteine-rich domain required for the multimerization of POSTN [169]. The cysteine-rich domain precedes a tandem of four homologous FAS1 domains (FAS1), which is responsible for the interaction of POSTN with integrins (see below) [170] and glycosaminoglycans [171]. The C-terminal hydrophilic domain binds with ECM proteins other than integrins (collagens, fibronectin, tenascin C, heparin, etc. [172]).

In cancer, POSTN contributes to EMT by organizing the resident structural proteins into an integral network [173]. Integrins are transmembrane heterodimeric receptors that mediate the interactions of cells and anchor the cells in the ECM [174]. By interacting with integrins (ανβ3, ανβ5, and α6β4), POSTN activates the intracellular signaling pathways with a wide range of biological effects from proliferation and survival to migration [175]. As transmembrane proteins, integrins are the molecular sensors that react to even minor changes in the cellular microenvironment that may influence cellular adhesion [176].

The interaction of POSTN with either integrin ανβ3 or α6β4 activates the PI3K/PKB signaling pathway and suspends apoptosis in the affected cells (Figure 2) [177]. Conversely, the exposure of cells with POSTN-specific antibodies significantly increases their apoptotic rate [178]. In turn, the binding of POSTN to integrin ανβ5 fuels the crosstalk with EGFR, promoting EMT in the affected cells [179]. In vascular endothelial cells, the interaction of POSTN with integrin αvβ3 upregulates the expression of FLK1/KDR (fetal liver kinase/kinase insert domain receptor), the specific receptor of VEGF, through the activation of FAK [180]. By increasing their sensitivity to proangiogenic growth factors VEGFA, -C, -D, and -E [181], POSTN stimulates the formation of capillary-like structures in vitro [182]. The latter suggests a similar role for POSTN in the neovascularization of tumors.

The overexpression of *POSTN* can alter the morphological characteristics of cultured cells. It can also increase their expression of mesenchymal biomarkers, such as VIM and fibronectin, indicating that POSTN contributes to the EMT of cancer cells [183]. Moreover, POSTN enhances the invasiveness of both primary cancer cells and transformed epithelial cell lines [184]. In addition, transplanting POSTN-overexpressing cancer cells into mice causes them to reorganize into rapidly growing neovascularized tumors [185].

POSTN has the potential to serve as a biomarker for predicting the invasiveness of cancer cells. Overexpression of POSTN is evident in various types of human cancers, including lung cancer and cancer cell lines [184]. According to Takanami et al. [186], 42% of NSCLC tumors are POSTN-positive. Moreover, the expression of POSTN correlates with tumor size, stage of the disease, invasion of lymph nodes by tumor cells, metastasis, and tumor recurrence [187]. The expression of POSTN in cancer cells increases in response to hypoxia [188]. Similar to LOXL2, patients with NSCLC have higher levels of POSTN in their blood serum, where this parameter correlates with invasiveness, metastasis, and poor survival [184]. In patients with advanced NSCLC, POSTN accumulates in the cytoplasm of tumor cells, suggesting the existence of non-canonical biological effects of POSTN associated with tumorigenesis. Published data also suggest that the level of POSTN indicates accelerated tumor progression, neovascularization, and lymphangiogenesis, as well as a worse prognosis for NSCLC patients [184].

### 5.8. P4HA1 and P4HA2

**Figure 5:**
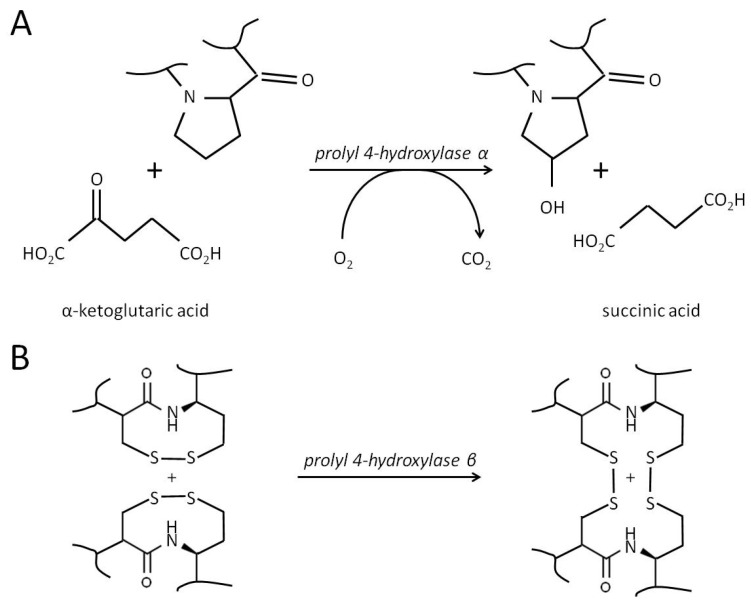
The enzymatic reaction catalyzed by prolyl 4-hydroxylase. (**A**). The hydroxylation of proline to 4-hydroxyproline by α subunits of P4H. For catalysis, the enzyme requires three different cofactors: molecular oxygen (O_2_), iron (Fe^2+^), and ascorbic acid (vitamin C). The essential proline residue of a substrate protein (e.g., “collagen”) shall be a part of the specific amino acid motif, typically -X-Pro-Gly-, where X can be any amino acid. During the catalytic process, α-ketoglutaric acid binds to the enzyme-Fe(II) complex, followed by a protocollagen strand and O_2_. After forming the ferryl ion and hydroxylation of the proline residue, the enzyme releases the nascent 4-hydroxyproline-containing polypeptide, CO_2_, and succinate. (**B**). The isomerization of disulfide bonds catalyzed by the β subunit of P4H. The catalytic process includes the formation of disulfide intermediates between the SH-groups of the substrate protein(s) and the enzyme (P4HB), their structural rearrangements to achieve the desired connectivity, and a recovery of the enzyme.

Prolyl 4-hydroxylase subunit α1 (P4HA1) is a part of prolyl 4-hydroxylase (P4H)/procollagen-proline dioxygenase [189,190]. The molecule of catalytically active enzyme is a heterotetramer composed of two identical α and two β subunits [191]. The human genome encodes four different α subunits of P4H, namely P4HA1, -A2, -A3, and -A4, and only one β subunit (P4HB). The names of P4H isoforms (P4H1, P4H2, P4H3, and P4H4) depend on what α-subunit they contain. The catalytic activities of α and β subunits are different. The αsubunit hydroxylates proline residues to 4-hydroxyproline targeting the specific -X-Pro-Gly- repetitive motif sequences in collagens (Figure 5A). This reaction is necessary to stabilize collagen triple helices, and it requires a preheteromerization of two α and two β into a tetramer. The monomer of P4HB exhibits disulfide isomerase activity (PDI)—Figure 5B. Dimerization of β subunits blocks the substrate-binding site and inhibits their catalytic activity [192]. In addition, the molecule of the β-subunit contains a signaling motif to retain P4H in the lumen of EPR. It prevents the relocation of the fully assembled enzyme to the cytoplasm.

The most prevalent α subunit, P4HA1, also hydroxylates proline residues in elastins, prion, argonaute 2 (AGO2), conotoxins, and other proteins that contain the -X-Pro-Gly- sequence [191]. In turn, one of its counterparts, P4HA2, hydroxylates proline residues in YAP1 (Yes-associated protein 1) [193], AGO1 [194], and mTOR [195]. The substrate specificity of P4HA3 and -4 is unclear, and their role in tumorigenesis is uncharacterized. Moreover, it is hard to say whether P4HA1 and -2 are capable of acting on substrate proteins of each other without a comparative analysis of their substrate specificities. On the other hand, P4HA1 and -A2 have distinct expression patterns [196,197]. This explains why P4HA1 is better characterized for its role in lung cancer, whereas P4HA2 is well known for its role in glioma [198,199] and cervical cancer [200].

P4HA1 and -A2 play an essential role in the three-dimensional folding of newly synthesized collagen [200]. However, P4HA1 is indispensable for this process, unlike P4HA2. A dysfunction of P4HA1 causes an autosomal dominant disorder presented with multiple abnormalities in connective tissues, such as a mild skeletal dysplasia without bone fragility, the hypermobility of joints, contractures, muscle weakness, and myopia [189]. Contrarily, its overexpression in malignant tumors indicates shorter overall survival and disease-free survival time for patients with various types of cancer [201,202].

Numerous studies show that *P4HA1* is upregulated in hypoxic conditions by the transcription factor HIF1 (e.g., [203]). Considering the role of P4HA1 in the posttranslational modification of collagens, it is worth speculating about the contribution of this enzyme to the neovascularisation of tumors and EMT. It also explains a tight control by miRNAs over the expression of P4H1. According to the published data, miR-122 [204,205], miR-124-3p in smooth muscle cells [206], and hsa-miR-124-3p in primary lung adenocarcinoma cells [201] target *P4HA1* mRNA for degradation. Moreover, the downregulation of hsa-miR-124-3p increases the ability of cultured A549 cells to invade.

P4HA1 influences the gene expression by stabilizing AGO2, the subunit of the main intracellular mRNAs-degrading machine, the ribonucleoprotein complex RISC (RNA-induced silencing complex) [207]. In RISC, AGO2 is responsible for binding to the guide strain of miRNA (guide RNA) [208]. This interaction activates RISC and directs it against mRNAs capable of hybridizing with the guide RNA. Interacting with AGO2, P4H1 hydroxylates its proline residue (Pro_700_), which is crucial for the stability of this protein and RISC [207]. Silencing P4HA1 or replacement of Pro_700_ with alanine by site-directed mutagenesis in human and murine cells in vitro reduces the stability of AGO2 and impairs the activity of RISC. In tumorigenesis, RISC is a necessary tool for changing the transcriptome of potentially malignant cells. Coordinating its actions with other genetic and epigenetic factors (transcription factors, non-coding RNAs, and chromatin-modifying enzymes), the post-P4H-modified AGO2 would facilitate EMT by accelerating the degradation of mRNAs encoding the epithelial proteins.

The numerous experimental studies also highlight the role of P4HA1 in tumorigenesis. Robinson et al. [201] demonstrate a significant increase in *P4HA1* mRNA in NSCLC tumors. Moreover, the upregulation of enzymes involved in the biosynthesis of collagen, including *P4HA1,* indicated a worse outcome for the patients. The other authors show the importance of P4HA1 for the clinical characteristics of the patients, such as overall survival and relapse-free survival [202,209]. The experiments on silencing *P4HA1* also confirm its crucial role in lung cancer growth, migration, and invasion. For instance, it is well-documented that P4H regulates the stability of HIF1α [210,211]. In turn, HIF1 promotes structural rearrangements in ECM, dissemination of tumor cells, and metastasis by upregulating *P4HA1* in CAF and tumor cells. In addition, the specific inhibitor of P4H, diethyl pythiDC decreases the malignancy of cultured lung cancer cells [201], suggesting that collagen-modifying enzymes, such as P4H, play a crucial role in controlling their invasiveness [195].

The role of P4HA2 in lung cancer deserves additional and more detailed studies. We know that P4HA2 stabilizes mTOR kinase by hydroxylating highly conserved proline residue (Pro_2341_) within the kinase domain of mTOR [195]. In this regard, the site-directed mutagenesis of Pro_2341_ for alanine slows the growth of LUAD cells and tumors. However, the hydroxylation of the essential proline residue is not the only way to activate mTOR. The alternative options include PI3K/PKB [212], WNT [213], and the MAPK-ERK [214] signaling pathways. Their role should not be underestimated, considering P4HA2 as a candidate biomarker for the invasiveness of cancer cells.

### 5.9. ITGA6

ITGA6 is the α_6_ subunit that is part of two integrin molecules: α_6_β_4_ and α_6_β_1_. Both integrins are cell surface receptors that mediate the interaction of neighboring cells to each other and their interaction with ECM proteins. These integrins play a role in maintaining the integrity of the cell membrane and also contribute to intracellular signaling by binding to laminins and netrin 4 [215]. The expression of *ITGA6* is under the control of various transcription factors. Some (e.g., AP1, HIF1, and MYC/myelocytomatosis oncogene) induce the gene, while others, like KLF9 and NFAT1 (nuclear factor of activated T cells 1), suppress it. *ITGA6* is one of the hypoxia-responsive genes and contains three HREs in its promoter [216]. As a result, the expression of *ITGA6* decreases when cells lack sufficient HIF1α.

The maturation of *ITGA6* pre-mRNA results in two transcripts, namely ITGA6A and ITGA6B [217]. These transcripts are different from each other on two C-terminal amino acid residues. This difference influences their affinity to the effector proteins and the ability to activate various signaling pathways. The expression of ITGA6A and ITGA6B is tissue-specific. Moreover, the tumor cells often express a shorter version of ITGA6, α_6_p, originated by proteolysis of the laminin-binding domain from the rest of the molecule. The truncated α_6_p remains integrated into the cellular membrane, where it functions as a decoy receptor.

After processing, *ITGA6* mRNA may interact with several miRNAs, namely miR-143-3p, miR-448, and miRNA-92a, which target it for degradation [215]. In turn, at least two lncRNAs may overrun their action. In ovarian cancer, OIP5-AS1 lncRNA prevents the repressive action of miRNA-92a. In salivary adenoid cystic carcinoma, ADAMTS9-AS2 lncRNA competitively binds to miR-143-3p.

Both α_6_β_1_ and α_6_β_4_ directly interact with protein kinases, such as the SRC family kinases (SFKs), FAK, and ILK (integrin linked kinase) [218]. These enzymes form multiprotein complexes that include integrins, cytoskeletal proteins, and signaling molecules to modulate the downstream signaling pathways. For instance, the binding of integrin α_6_β_4_ to laminins activates RAC1 (Rac family small GTPase 1), PKC (protein kinase C), PI3K, and ERK signaling pathways, which regulate the proliferation, survival, and migration of tumor cells [215]. The named integrins also interact with tyrosine kinase receptors (e.g., EGFR-ERBB2, MST1R/macrophage stimulating 1 receptor, and c-MET/methoprene-tolerant) and their ligands, helping to amplify the oncogenic signaling in tumor cells [215,219]. For example, α_6_β_4_ improves the recognition of IGF1 by its specific receptor IGF1R by interacting with both of them [220]. In this respect, when cells attach to ECM, IGF1 has a minimal effect on intracellular signaling. However, IGF1 strongly induces intracellular signaling in an α_6_β_4_-dependent manner if cell-to-matrix adhesion is low.

The role of ITGA6 in maintaining cell stemness is well-documented. Using ITGA6 as a biomarker for cancer invasiveness could be advantageous. Being readily detectable in more than 30 adult stem cells and cancer stem cells (CSCs), including glioblastoma [221], breast [216], colon [222], and prostate cancers [223], ITGA6 is present at low or undetectable levels in healthy tissues. Specifically, knocking *ITGA6* down in lung cancer cells decreases their invasiveness [224]. In lung tumors, the expression of ITGA6 increases in response to the stiffening of ECM and hypoxia. Higher levels of ITGA6 correlate with tumor development, aggressiveness, increased risk of recurrence, poor patient prognosis, shorter event-free survival, and overall survival time in cancer patients [215].

There is a way to minimize a possible bias caused by the different biological effects of α_6_β_1_ and α_6_β_4_ integrins by substituting ITGA6 with ITGB4 at the protein level. This approach seems reasonable since ITGA6 is the only α subunit capable of forming a complex with β_4_ integrin. For example, a lung cancer tissue microarray conducted by Stewart et al. [225] indicates specifically a higher expression of integrin β_4_ in lung SCC compared to LUAD tumors, suggesting a greater clinical significance of the α_6_β_4_ integrin for lung SCC. Moreover, they found a positive correlation between the expression of ITGB4 and venous invasiveness of cancer cells, as well as negative correlations with reduced OS and EFS in both groups of patients. Based on the results of their analysis, the authors recommend ITGB4 rather than ITGA6 as an adverse prognostic biomarker for lung cancer.

To achieve more with ITGA6 as a biomarker, it would be reasonable to make it a part of a molecular signature that discriminates the invasive lung cancer cells from lung metastasis originating from different types of cells. Presumably, such composite biomarkers will also tend to describe more accurately the changes in tumor cells and their phenotype and. In addition, they will provide valuable information on the origin of the cells [226,227]. For instance, Wang et al. [226] propose a three-integrin score (TIS) based on the expression of ITGA5, ITGA6, and ITGAL. Analyzing the data of LUAD and lung SSC patients presented in The Cancer Genome Atlas (TCGA), the authors discovered a worse prognosis for LUAD patients with higher TIS in the early stages of the disease. They also proposed that the possible role of ITGA5 in the suggested triad was mainly associated with cell migration and invasion, whereas ITGAL was responsible for recognition and killing cancer cells. However, they did not speculate about the role of ITGA6. In another study, Herreros-Pomares et al. [227] proposed an alternative three-gene signature of *CDKN1A* (cyclin dependent kinase inhibitor 1A), *SNAI1*, and *ITGA6* (the CSC score) for assessing OS of patients with LUAD and lung SCC.

### 5.10. ASAP1

ASAP1 (ADP-ribosylation factor GTPase-activating protein with SH3 domain, ankyrin repeat, and PH domain 1) is a cytoskeletal protein. ASAP1 contributes to cell migration by reorganizing filamentous actin-based structures, such as stress fibers, focal adhesions, and circular dorsal ruffles (CDRs) [228]. Due to myristylation, ASAP1 is anchored in the cellular membrane, bringing together its binding partners and filamentous actin. Since ASAP1 is a GTPase-activating protein, it inactivates small GTPases, namely ARF1 and ARF5 (ADP-ribosylation factors 1 and 5, respectively) [229,230], promoting the hydrolysis of GTP. For instance, interacting with ARF1-GTP, ASAP1 converts it into the inactive form (ARF1-GDP). In turn, the inactivation of the named small GTPases improves the recycling of CDH2 back to the cell surface, shifting the balance of E and N cadherins in favor of the endothelial cadherin. Moreover, ASAP1 participates in the recycling of ITGB1 through its interaction with protein kinase D2 (PRKD2). Binding and phosphorylating ASAP1, PRKD2 increases its affinity to ITGB1. Then, the complex of PRKD2 and ASAP1 binds to ITGB1, takes it out of the endosome, and brings it back to the cellular membrane [231].

ASAP1 has several binding partners. Directly binding to FAK, ASAP1 contributes to the formation of focal adhesions. Silencing ASAP1 interferes with their maturation, disrupts stress fibers, and inhibits the recruitment of MYH9 (myosin 9/nonmuscle myosin 2) and F-actin. It also causes the dissociation of paxillin and FAK [229]. Moreover, ASAP1 interacts with myosins, actin-associated motor proteins [228,230]. For instance, the interaction of ASAP1 and MYH9 is critical for cell migration. MYH9 catalyzes the hydrolysis of ATP and uses the generated energy to force the cell vesicles to move across the cytoskeletal filaments. It forms the contractile structures to retract the trailing end of cells when the cell is moving. Similar MYH9-containing structures participate in cell division.

Along with other proteins, ASAP1 regulates the formation of CDRs, circular actin-rich structures appearing in the cell in response to external stimulation during the internalization of cell receptors and migration. Silencing ASAP1 facilitates the formation of CDRs. Contrarily, its overexpression inhibits this process [230]. The biological effect of ASAP1 on CDRs also depends on MYH9. Moreover, ASAP is necessary for the functioning of podosomes, conical, actin-rich structures associated with the outer side of the cellular membrane. In the cell, podosomes play a dual role. They participate in the cellular movement and degradation of ECM. The core of the podosome accumulates F-actin and actin-regulatory molecules surrounded by a ring of adhesion and structural proteins. Silencing ASAP1 inhibits the formation of podosomes. To participate in the formation of podosomes, ASAP1 does not have to be catalytically active. However, it has to be phosphorylated. Contrarily, the overexpression of ASAP1 has no detectable effect on podosomes [229].

The direct involvement of ASAP1 in the formation and functioning of subcellular structures, like CDRs and podosomes, and the reorganization of stress fibers, makes it a candidate biomarker for cancer invasiveness. In other words, the main biological effects of ASAP1 favor the invasive cancer cells to migrate. It is well documented that when binding to cortactin (CCTN), ASAP1 promotes the rearrangements of the cytoskeleton, such as a polymerization of actin filaments and their bundling [232,233,234]. ASAP1 also rearranges bilayers of phospholipids, facilitating their integration and reintegration into the cellular membrane [235]. By connecting bending membranes with polymerizing actin, ASAP1 controls the dynamic of invadopodia [236]. However, the ability of ASAP1 to distinguish invasive and rapidly proliferating tumor cells is not evident because of its direct participation in cell division. Moreover, using *ASAP1* as a sole biomarker of invasive lung cancer would be restricted due to a broad overexpression in many other malignancies (rev. [237]). In other words, staining for ASAP1 cannot discriminate invasive lung cancer cells and cells from metastatic lung lesions.

### 5.11. GPRIN1

GPRIN1 (G protein regulated inducer of neurite outgrowth 1) is a poorly characterized protein found in the cellular membrane and synapses. This protein plays a role in regulating the signal transduction and Ca^2+^ homeostasis. Mechanistically, GPRIN1 interacts with G_αo_, -GTP, GAP43 (growth associated protein 43), and CDC42 (cell division cycle 42), forming a protein complex. This complex enables α7 nicotinic receptors to increase the flow of Ca^2+^ and promote the reorganization of the cytoskeleton to establish the early neuronal network and support the functioning of mature neurons [238].

Another binding partner of GRIN1, SPRY2 (sprouty receptor tyrosine kinase signaling antagonist 2), inhibits MAPK signaling. Both SPRY2 and G_αo_ compete for the same binding site in the molecule of GRIN1. The activation of the G_o/i_-coupled CB1 receptor shifts the balance between GRIN1-G_αo_ and GRIN1- SPRY2 toward GRIN1- SPRY2. It increases the phosphorylation of SPRY2 and attenuates the activation of MAPKs. The overexpression of GRIN1 prevents phosphorylation of SPRY2 and potentiates the activation of the MAPK signaling pathway [239]. In turn, the suppression of GRIN1 neutralizes both G_αo_ and SPRY2.

In healthy individuals, the expression of GPRIN1 is evident in the specific areas of the central nervous system (CNS) and peripheral nerve system. Moreover, GPRIN1 is not among the proteins broadly overexpressed in various cancers. The pan-cancer analysis of the data deposited to the TCGA database performed by Zhou et al. [240] revealed the upregulation of GPRIN1 in renal papillary cell carcinoma and LUAD tumors. The other authors also report [241] a downregulation of GPRIN1 in gastric cancer (tissues and cells) and higher expression of GPRIN1 in breast cancer [242] using the data deposited in the same database.

The analysis of original clinical data conducted by Zhuang et al. [243] confirmed a higher expression of *GPRIN1* in LUAD compared to healthy lung tissue. The authors also discovered that the expression of GPRIN1 correlated with OS and poor prognosis for patients. Through Cox regression analysis, they demonstrated that GPRIN1 can be an independent predictive factor for LUAD. At the same time, their in vitro experiments produced less unequivocal results. Specifically, Zhuang et al. found that the expression of *GPRIN1* positively correlated with the invasiveness of A549 cells, while it had the opposite effect in lung epidermoid carcinoma Calu-1 cells. Due to this inconsistency, further studies, such as a recent one conducted by Wang et al. [244], are necessary to elucidate the genetic basis of lung tumors in which invasiveness does not align with the expression level of *GPRIN1*.

Although the role of GPRIN1 in invasiveness requires further clarification, a recent study conducted by Mao et al. explains the details of the mechanism responsible for the upregulation of *GPRIN1* in LUAD [245]. Using the sequencing methods, the authors compared the patterns of methylated adenine residues (m6A) in mRNAs of three LUAD tumors and paired samples of adjacent lung tissues. They discovered that cancer cells regulate the expression of *GPRIN1* mRNA with epigenetic mechanisms. Specifically, the authors found *GPRIN1* mRNA among the twenty most methylated mRNAs detectable in tumor samples. Then, they discovered a correlation in the expression of GPRIN1 and YTHDF1 (YTH N6-methyladenosine RNA binding protein 1), a protein that promotes the export of mRNAs from the nucleus to the cytoplasm and the following translation after a ribosome recognizes m6A. Since these data suggested a faster processing and more efficient transcription of *GPRIN1* mRNA, the authors sequenced the transcriptomes and confirmed *GPRIN1* among the most transcribed genes in LUAD samples. In addition, they also found that m6a residues predominantly reside in the 3′-UTR region of *GPRIN1* mRNA and proposed that methylation also improves the stability of *GPRIN1* mRNA by delaying its degradation.

### 5.12. ABL2

ABL2/ARG (Abelson murine leukemia viral oncogene homolog 2/Abelson-related gene) is one of two non-receptor ABL kinases encoded in the human genome [246]. In the cell, ABL2 localizes to the cytoplasm and preferentially gathers in the peripheral subcellular structures enriched with F-actin, adherens junctions, phagocytic cups, and invadopodia [247]. The alternative splicing of *ABL2* mRNA produces multiple protein-coding isoforms. One of them (the isoform 1b) contains the N-terminal glycine susceptible to myristoylation, suggesting its possible anchoring to the cellular membrane [248].

The existence of multiple mechanisms controlling the kinase activity by posttranslational modifications, inhibitors (e.g., PIP_2_), and binding partners suggests that ABL2 plays a crucial role in cellular signaling. For instance, the interaction of ABL2 with other proteins may result in significant conformational changes and cause the transition of ABL2 from the closed to open (catalytic active) state. The following phosphorylation by one of the upstream tyrosine kinases stabilizes the catalytically active ABL2 by preventing its reverse transition to the closed conformation. The phosphorylation also allows the enzyme to achieve the proper orientation of its catalytic site to become capable of activating the downstream signaling pathways [249].

ABL2 plays a critical role in influencing the invasiveness of tumor cells by reorganizing their cytoskeleton. It directly binds F-actin, bundling the actin filaments with microtubules and accelerating the polymerization of actin filaments by activating effector proteins [250]. A faster polymerization provides the protrusive force at the leading edge of the moving cell to push the membrane forward. After the protrusion of the membrane, integrins mediate cell adhesion to ECM, which can either turn over or enlarge into mature focal adhesions through the action of the RHOA GTPase (Ras homolog family member A GTPase). Silencing *ABL2* restricts cell motility by inhibiting the formation of protrusions and invadosomes [251].

The dysregulation of ABL2 occurs in various cancers [246]. One of its causes in T-cell acute lymphoblastic leukemia is the chromosomal rearrangements producing the ETV6-ABL2 fusion gene (ETV6—ets variant 6) with a constitutively active tyrosine kinase domain [252]. In solid tumors, ABL2 may acquire gain-of-function mutations (Gly_1158_→Ser, Ala_234_→Val, etc.) or amplify the number of copies in the genome. To date, numerous studies have explored the role of ABL2 in the invasiveness of tumor cells. For instance, Gil-Hennet al. [253] proved the role of ABL2 in the invasiveness of human breast cancer cells. The authors found that *ABL2*-deficient tumors are significantly less invasive compared to ones with non-altered expression of ABL2. Despite this, the size of examined tumors was larger, suggesting a higher proliferation rate of their constituting cells. In addition, the expression of genes associated with cell proliferation was higher, whereas one of the genes participating in cell motility was lower compared to tumors with normal levels of ABL2.

Contrarily, the expression of *ABL2* in many other tumors is not significantly higher compared to the surrounding areas of the same organ. The main reason for a relatively low *ABL2* could be the control by miRNAs (miR-30a-5p—in LUAD [254], miR-4723—in prostate cancer [255], miR-425-5p—in esophageal squamous cell carcinoma, lung and colon cancer [256], etc.) targeting *ABL2* mRNA. Alternatively, the suppression of ABL2 may occur at the protein level. In this regard, De Marco et al. reported a negative feedback loop controlling the level of ABL2 in clear cell renal cell carcinomas [257]. Comparing the tumors of higher and lower grades, the authors discovered activation of the TGFβ1/SMAD signaling in advanced tumors (SMAD—mothers against decapentaplegic homolog 2). Analyzing the changes in the expression of *ABL2* and the intensity of TGFβ1/SMAD signaling, they found a negative correlation between these parameters. Moreover, cells stimulated with TGFβ1 (transforming growth factor β1) increased ubiquitination and degradation of ABL2 and the production of ROS. At the same time, uncontrolled expression of *ABL2* promoted the invasion and maturation of invadopodia in tumor cells.

After all, the published data indicates that the proportion of tumors expressing high levels of ABL2 is relatively low, typically not exceeding 1–2%. In NSCLC tumors, the corresponding percentage is less than 0.5% [258]. The chances of finding an occasional ABL2 mutation in a solid tumor and an NSCLC tumor are higher, while they also do not exceed 20–35% [247]. Consequently, it would be more reasonable to consider ABL2 for a panel of genes indicating cancer invasiveness rather than using it as an individual biomarker to characterize cell invasiveness in a randomly chosen lung tumor.

### 5.13. PLK4

PLK4 (polo-like kinase 4) is a serine-threonine kinase that predominantly gathers at the centriole [259]. The upregulation of *PLK4* may cause the amplification of the centrosome, whereas the downregulation of PLK4 leads to its loss. The loss of centrosome in a non-cancer cell prevents the cell from passing through the G_1_/M checkpoint and causes a cell cycle arrest. However, if the cell does not control the cell cycle, the amplification of the centrosome leads to changes in the cell karyotype and genomic instability.

The expression of PLK4 is under the control of several mechanisms. At the transcriptional level, the transcription factors, such as ARF6, C/EBPβ (CCAAT enhancer binding protein β), and KLF14, suppress *PLK4*. The other transcription factors (e.g., NFκB and E2F) induce it [260]. Moreover, the transcription of *PLK4* is cell-cycle dependent. PLK4 is undetectable during the interphase (G_0_) and at the beginning of G_1_. Then, its expression increases and reaches the maximum during mitosis. After that, the level of PLK4 sharply declines.

Before the translation, *PLK4* mRNA may become a target of miRs (e.g., miR-126 and miR-338-3p), which cause its degradation and prevent translation of the mRNA on the ribosomes. After translation, PLK4 exhibits a low catalytic activity because part of its molecule blocks the phosphorylation site. Phosphorylation is necessary for the activation of PLK4. To make the phosphorylation sites accessible, PLK4 has to homodimerize [259,260]. The homodimers of PLK4 phosphorylate themselves and play the role of substrates for some protein kinases (MAP3K1/mitogen-activated protein kinase kinase kinase 1, MAP3K4, and MAP3K7). To reverse phosphorylation, PLK4 has to interact with the phosphatase 2A (PP2A). The phosphorylation destabilizes PLK4, whereas dephosphorylation improves its stability.

The phosphorylated PLK4 gains the maximal catalytic activity. However, it also becomes a desirable target for E3 ubiquitin ligases (SCFβ-TRCP E3 ubiquitin ligase complex or MIB1—mindbomb 1). The ligases recognize PLK4 and mark it with ubiquitin. Then, the ubiquitinated PLK4 can either degrade in proteasome or become deubiquitinated by CYLD (cylindromatosis) and recycled. In addition, the molecule of PLK4 contains two sites of acetylation. The acetylated PLK4 is present in the centrosome during the transition of the cell from the G_1_ to S phase of the cell cycle. According to Fournier et al. [261], it is necessary to prevent the amplification of the centriole by reducing the kinase activity.

Considering the pros and counterarguments of using PLK4 as a biomarker of cancer invasiveness, we have to admit that numerous studies report the overexpression of *PLK4* in different human cancers. Their authors also suggest correlations between the expression of PLK4, tumor stage, and poor prognosis for patients (e.g., [262]). Some other authors also acknowledge that PLK4 also facilitates the invasiveness and metastasis of tumor cells via an activation of the ARP2/3 complex (ARP—actin-related protein) and small GTPases RHO and RAC [263,264,265] despite its a well-documented role in the cell cycle.

According to one such paper, PLK4 interacts with the ARP2/3 complex that initiates branching of the actin filaments. [266]. Then, PLK4 phosphorylates ARP2, allowing its interaction with nucleation-promoting factors (NPFs), like WASP (Wiskott–Aldrich syndrome) or WASF1 (WASP family, member 1). These factors increase the affinity of the ARP2/3 complex to the filamentous actin. They also deliver actin to the ARP2/3 complex and promote their interaction. Following the dissociation of NPFs, the new branch starts growing by recruiting more actin monomers. Their growth facilitates the formation of new subcellular structures, like lamellipodia, at the leading edge [263]. In support of these findings, the reduced phosphorylation of ARP2 by PLK4 inhibits the reorganization of the actin cytoskeleton [267]. PLK4 also phosphorylates ECT2 (epithelial cell transforming 2), which, in turn, activates RHO GTPase to promote cell migration via the formation of focal adhesions [265]. The overexpression of PLK4 in MCF10A cells leads to the activation of RAC, which promotes invasion and metastasis by weakening the cell adhesion and causing the formation of membrane ruffles [264]. Summarizing the above data, we assume that the use of PLK4 to assess the invasiveness of cancer cells needs to rely on either high-quality imaging analysis or cell sorting due to the necessity to distinguish two different phenotypes: rapidly proliferating epithelial and slow proliferating motile mesenchymal-like cells colocalized in the same tumor.

## 6. Limitations and Perspectives of Biomarkers Research

Although biomarker research has the potential to significantly advance our understanding of diseases and improve diagnostics, some limitations and challenges are associated with this field. One major limitation is the high-dimensional data produced in proteomic studies, suggesting thousands of potential biomarkers often considered independent. This approach ignores plausible causal relationships (signal–target, transcription factor–coactivator or corepressor, monomer–heteromer, etc.). Neglecting causal relationships may have far-reaching negative consequences, such as shifting attention to secondary effects rather than focusing on the driving forces of a specific condition. In this regard, similar studies conducted by several teams using alternative technologies (SOMAscan, Proximity Extension Assay (PEA), etc. [268]) are highly desirable. In addition, various improvements to individual studies, such as increasing the sample size and the number of parallel experiments and using corrections for multiple testing, can also reduce the false discovery rate [269]. Another aspect of the same problem is individual differences in genetics, environment, and lifestyle that influence the expression of a desired biomarker, making it difficult to establish universal thresholds for what constitutes a “normal” or “abnormal” level. Several options, such as performing multivariate analysis (MANOVA), stratification of the data, and employing hierarchical models, would be reasonable to address variability in personal data and minimize its impact.

At the same time, some studies may still use biomarkers of general poor health (inflammation, metabolic irregularities, benign tumors, etc.) to characterize the health status of patients with cancer. The reasons for this disadvantage could be either poor knowledge or misrepresentation of biomarkers associated with specific conditions. Additionally, narrowing the input (e.g., using idealized cohorts of patients in machine learning [270]) and randomizing the selection of potential indicators will likely overstate their role as biomarkers of specific conditions. The latter will lead to misinterpretations of the results and misdiagnoses of patients. Moreover, it will allocate necessary healthcare resources from individuals who need them the most, potentially delaying appropriate care. At the same time, treatment of misdiagnosed individuals with anticancer therapy will likely cause complications and side effects that could be avoidable in the first place. Thus, focusing on general health biomarkers may overshadow the specific biomarkers that would be more relevant for accurate diagnosis and treatment of cancer. In addition, if a biomarker does not participate in a core biological process of the disease, there is a chance that it represents an epiphenomenon and may not accurately reflect changes in the pathogenesis.

Bridging the gap between proteomics research and clinical applications is a significant challenge. In recent years, proteomics has made substantial progress in the reproducibility and accuracy of quantitation results. However, it continues to face numerous technical problems around the identification of proteoforms, the solubility of proteins, mass spectrometry dynamic range, and the complexity of the proteome. Recently, significant improvements have made it possible to confront these challenges. The new generation of solubilizers, such as Azo and modular surfactants, provide an opportunity to extract membrane and ECM proteins from tissue samples and, if possible, protect their structural integrity [271,272]. The invention of surface-functionalized multivalent superparamagnetic nanoparticles [273,274] and aptamers [268] improved the enrichment of samples with phosphoproteins and low-abundance proteins, respectively. The gas and liquid chromatographs underwent further optimizations to tackle the problems associated with the proteome complexity. In turn, the modified equipment for capillary electrophoresis enabled coupling to mass spectrometers. Adding new chemistries for liquid chromatography to the pipelines significantly enhanced the separation efficiency [275].

Several new proteomic technologies, such as Simoa^®^ (Quanterix Corporation, Billerica, MA, USA) and Luminex xMAP (Luminex Corporation, Austin, TX, USA), represent variations of digital Enzyme-Linked Immunosorbent Assay (ELISA) [35]. They have impressive sensitivity. For instance, Simoa^®^ [276,277] can recognize single molecules of protein biomarkers per sample (10^−18^ g/mL) and six different biomarkers per load. Luminex xMAP [278] is less sensitive. However, it identifies a hundred biomarkers per run in the same sample with an impressive speed of over 4800 plates per hour. At the same time, these new technologies are more suitable for large-scale industrial projects in pharmaceutical research rather than for routine lab work in a local hospital.

Unlike experimental approaches presented by competitors, the PEA technology (Olink Proteomics, Uppsala, Sweden) [279,280] appears more beneficial for a regular lab due to its modular structure. The initial steps of the experiment are similar to an immunoassay, while the later ones resemble sequencing of regular DNA samples. Instead of antibodies, this method uses the conjugates of antibodies with oligonucleotides. Each pair of antibodies is specific to one of the biomarkers, and each pair of oligonucleotides carries a unique barcode sequence and has a short complementary sequence at the 3’-end that lets them form a duplex. During sample processing, the sequencing machine identifies, quantifies, and interprets the encoded barcodes, revealing the composition of each sample. Since the lab personnel have to use the equipment more efficiently, this technology has the potential for future development and integration with other lab tools. Moreover, the facility using this technology may remain at its current scale.

Although existing technologies for detecting protein biomarkers are constantly improving, a better experimental approach has yet to come. This approach will combine selectivity and high affinity of antibodies with the power of microarrays. It must distinguish separate proteoforms and provide reliable results even in denaturing conditions. In the future, existing technologies may meet these demands after undergoing a series of modifications and upgrades. However, after carefully reading this review paper, the reader will likely agree that the consumer’s choice of the most satisfying technology to detect protein-based biomarkers in a free-market economy will always be subjective due to competition and alternative solutions offered by different private enterprises.

Technological advances in proteomics come side-by-side with the development of new experimental models. The creation of new experimental models, particularly three-dimensional (3D) culture models, has revolutionized the field of biomarker research [281,282,283]. These models, spheroids and organoids, offer several advantages over traditional two-dimensional (2D) monolayered cultures previously used by the industry for screening candidate drugs, including more accurate mimicry of complex cell interactions and tissue architecture. Three-dimensional models promote cell differentiation and maturation and can remodel their microenvironment by depositing extracellular matrix proteins, mimicking the parental tumor. Moreover, they preserve the cell composition and the specific surface biomarkers. At the same time, the long time required for their formation and growth, typically several weeks or months, is the principal limitation. Numerous methods are available to produce 3D models, including scaffold-free and scaffold-based techniques, and the choice of method can affect the external properties of the models [284]. Respectively, further studies would standardize the existing protocols and adjust them to specific needs.

The scientific community is making significant strides in addressing the existing challenges in biomarker research. By leveraging various approaches, researchers are paving the way for a deeper understanding of biological systems, enabling the development of novel diagnostic and therapeutic applications. As a result, the field of biomarker research is advancing rapidly, and novel breakthroughs are emerging to hold great promise for improving human health.

## 7. Conclusions

Advancements in molecular studies have improved our understanding of cancer, offering new tools for early detection of lung cancer and determining the most suitable treatment strategies for patients. Identifying the specific protein biomarkers associated with the invasive phenotype of cancer cells is critical to predicting the behavior of potentially malignant tumors. The high blood flow in the lungs and the lack of effective early diagnostic biomarkers for lung cancer increase the likelihood of the disease spreading to lymph nodes and other parts of the body. An accurate diagnosis of cancer invasiveness requires the study of several cell type-specific genes. Conventional methods, such as PCR and immunohistochemistry, are advantageous due to their widespread availability in medical labs, offering the opportunity to optimize the selection of molecular biomarkers for monitoring the disease over time.

We believe the proposed protocol for identifying invasive cancer will satisfy highly populated countries and countries with low income. It is expected that this protocol will use a minimal panel of molecular targets to confirm the origin of cancer cells. In the case of immunochemistry, the panel may include tissue-specific targets, such as KRT7, KRT20, and TTF1. In addition, lab work will identify biomarkers specific for a broader spectrum of cells like LOX, LOXL2, and MAPK15 to reveal cells with invasive phenotypes in the primary tumor and circulation. The accurate assessment of their expression will help to select the most appropriate treatments because some of these biomarkers can also be potential targets for anticancer therapy.

The recently proposed non-invasive and minimally invasive techniques offer earlier and faster cancer detection with reduced risk. This trend in biomarker research focuses on finding suitable biomarkers in exhaled breath and body fluids, like blood and saliva. In addition to the proteins secreted by invasive cells (see examples in Section 5), several new groups of biomarkers, such as circulating cell-free tumor nucleic acids (DNA or RNA), circulating microRNAs, and circulating exosomal proteins, are under consideration. An example of such a technique is the CancerSEEK test, which detects eight circulating proteins and sixteen genetic mutations in blood [285]. The MCED (multi-cancer early detection) test explores a unique methylation pattern of cell-free DNA, also known as circulating tumor DNA (ctDNA). By analyzing the changes in this pattern, it is possible to identify cells that have originated this DNA and the form of the cancer. MCED uses next-generation sequencing (NGS) to access this pattern and identify 50 types of cancers [286].

Cancer causes significant changes in cell metabolism and production of unique side metabolites, such as fragments of cellular lipids and multicarbon organic acids referred to in the literature as VOCs (volatile organic compounds). These compounds have a different representation in body fluids, depending on the form of cancer and stage of the disease. Some of them are detectable in the exhaled breath of lung cancer patients using gas chromatography and LC-MS [287]. Although this method is highly accurate in diagnosing breast and other cancers, it has not yet received approval from the FDA or EMA. In the future, this and other similar methods based on omics techniques will revolutionize cancer detection, making them accessible to the entire population. These methods will enable early detection of lung cancer and provide a more accurate way to classify tumors, assess clinical outcomes in patients with metastatic disease, and detect residual disease if it persists [288].

## Figures and Tables

**Table 1:** Prospective protein biomarkers for invasive lung cancer.

Biomarker	Full Name	UniProt ID
KRT7	Keratin, type II cytoskeletal 7	https://www.uniprot.org/uniprotkb/P08729/entry (accessed on 22 August 2024)
KRT20	Keratin, type I cytoskeletal 20	https://www.uniprot.org/uniprotkb/P35900/entry (accessed on 22 August 2024)
TTF1	Thyroid transcription factor 1	https://www.uniprot.org/uniprotkb/P43699/entry (accessed on 22 August 2024)
LOX	Protein-lysine 6-oxidase	https://www.uniprot.org/uniprotkb/P28300/entry (accessed on 22 August 2024)
LOXL2	Lysyl oxidase homolog 2	https://www.uniprot.org/uniprotkb/Q9Y4K0/entry (accessed on 22 August 2024)
MAPK15	Mitogen-activated protein kinase 15	https://www.uniprot.org/uniprotkb/Q8TD08/entry (accessed on 22 August 2024)
POSTN	Periostin	https://www.uniprot.org/uniprotkb/Q15063/entry (accessed on 22 August 2024)
P4HA1	Prolyl 4-hydroxylase subunit α1	https://www.uniprot.org/uniprotkb/P13674/entry (accessed on 22 August 2024)
P4HA2	Prolyl 4-hydroxylase subunit α2	https://www.uniprot.org/uniprotkb/O15460/entry (accessed on 22 August 2024)
ITGA6	Integrin, subunit α6	https://www.uniprot.org/uniprotkb/P23229/entry (accessed on 22 August 2024)
ASAP1	Arf-GAP with SH3 domain, ANK repeat and PH domain-containing protein 1	https://www.uniprot.org/uniprotkb/Q9ULH1/entry (accessed on 22 August 2024)
GPRIN1	G protein-regulated inducer of neurite outgrowth 1	https://www.uniprot.org/uniprotkb/Q7Z2K8/entry (accessed on 22 August 2024)
ABL2	Abelson murine leukemia viral oncogene homolog 2	https://www.uniprot.org/uniprotkb/P42684/entry (accessed on 22 August 2024)
PLK4	Polo-like kinase 4	https://www.uniprot.org/uniprotkb/O00444/entry (accessed on 22 August 2024)
